# 嵌合抗原受体T细胞联合治疗策略在淋巴瘤中的研究探索

**DOI:** 10.3760/cma.j.issn.0253-2727.2022.10.014

**Published:** 2022-10

**Authors:** 寅嫱 张, 恒 梅, 豫 胡

**Affiliations:** 华中科技大学同济医学院附属协和医院血液科，湖北省肿瘤疾病细胞治疗临床医学研究中心，武汉 430022 Institute of Hematology, Union Hospital, Tongji Medical College, Huazhong University of Science and Technology, Hubei Clinical Medical Center of Cell Therapy for Neoplastic Disease, Wuhan 430022, China

嵌合抗原受体T细胞（CAR-T细胞）在治疗复发难治性B细胞淋巴瘤患者中具有显著优势，自2017年Yescarta首次获批以来，已有6款可用于B细胞淋巴瘤的CAR-T产品上市。然而，部分患者在接受CAR-T治疗后，仍然出现耐药、复发的情况，其与用于制备的T细胞状态不良、抑制性免疫微环境、输注后炎症反应、患者治疗前存在高肿瘤负荷等导致的CAR-T细胞体内扩增困难和加速耗竭相关。小分子药物、抗体药物、放疗及造血干细胞移植作为抗肿瘤治疗的一部分，在直接杀伤肿瘤细胞的同时，对免疫微环境，特别是T细胞的活性、增殖、表型都有不同程度的调节作用。本文我们将小分子药物、抗体药物、放疗和造血干细胞移植与CAR-T细胞联合的相关基础研究和临床试验进展综述如下。

一、CAR-T细胞联合药物治疗

（一）激酶抑制剂

1. 布鲁顿酪氨酸激酶（BTK）抑制剂：BTK是一种连接B细胞受体信号、Toll样受体信号和趋化因子受体信号的关键分子，促进细胞增殖、抗体分泌以及细胞因子产生。伊布替尼作为第一代BTK抑制剂，具有更强的靶细胞结合力和更广泛的免疫微环境调节效应，可以通过转化细胞亚群、降低免疫检查点表达、诱导免疫反应方向等多种途径显著提升CAR-T细胞的杀伤功能。同时作为趋化因子受体下游分子，BTK抑制剂可以通过调节趋化和黏附促进肿瘤细胞向外周血迁移。BTK抑制剂可抑制髓系来源的抑制细胞的生成、迁移，从而减少TNF-α和活性氧（ROS）的产生，减少免疫负向调节。

采集细胞前应用伊布替尼可以改善体内T细胞状态，有效提升CAR-T细胞的疗效。慢性淋巴细胞白血病（CLL）和小淋巴细胞淋巴瘤（SLL）患者由于长期暴露在抗原刺激下，体内T细胞适应性下降，分化程度较低的类幼稚细胞比例下调，所制备的CAR-T细胞具有较低的扩增和存续能力。尹青松等[1]报道了1例CLL伴TP53突变患者口服伊布替尼5周后联合CD19 CAR-T细胞治疗，回输后1个月达完全缓解。Gauthier等[2]和Siddiqi等[3]的临床试验结果显示，伊布替尼联合CAR-T治疗后CLL/SLL患者的客观缓解率分别为83％和95％。Chavez等[4]对比弥漫大B细胞淋巴瘤（DLBCL）患者中单采前后应用伊布替尼的疗效差异，证实单采前应用的患者总有效（OR）率和完全缓解（CR）率显著提高；ZUMA-2的回顾性分析显示MCL患者在单采前使用伊布替尼较阿卡替尼可获得更好的疗效（65％对40％）[5]。

伊布替尼联合治疗存在相对可控的不良反应。在CLL/SLL的临床试验中，仅1例出现3级细胞因子释放综合征（CRS），3级及以上免疫效应细胞相关神经毒性综合征（ICANS）的发生率分别为16％和26％；在套细胞淋巴瘤（MCL）的临床试验中，伊布替尼组CRS和ICANS发生率高于阿卡替尼组。值得注意的是，伊布替尼相关心律失常导致1例患者死亡。

目前临床试验的用药剂量参考伊布替尼的标准剂量，治疗CLL/SLL推荐420 mg/d，MCL推荐560 mg/d。如果采用自体T细胞制备CAR-T细胞，建议患者至少单采前2周至1个月开始服用BTK抑制剂，并在CAR-T细胞可监测期间持续使用。

2. 酪氨酸激酶抑制剂（TKI）：TKI通过干扰淋巴细胞特异性蛋白酪氨酸激酶的合成抑制 CD3ζ和T细胞受体相关蛋白激酶70kDa（ZAP70）ζ链的磷酸化，限制活化T细胞核因子（NFAT）的诱导功能，从而减少CAR信号下行。Mestermann等[6]和Weber等[7]从多种TKI抑制剂中筛选出达沙替尼作为潜在有效的药物，通过细胞和小鼠体内实验证实其可逆性暂停CAR-T细胞对肿瘤细胞的溶解作用，减少IFN-γ、IL-2、GM-CSF、TNF-α等细胞因子的释放，并且在移除后不影响CAR-T细胞的继续增殖和活化，提示短疗程的达沙替尼可以作为CAR-T“开关”，在不影响疗效的基础上，预防致死性CRS的发生。

基于以上研究，调整达沙替尼的剂量和使用时机可以通过表观遗传重构抑制CAR-T细胞分化和衰竭，增强其抗肿瘤能力[8]–[9]。研究发现经浓度为30 nmol/L达沙替尼处理后的CD28/CAR-T细胞具有更加幼稚的表型，表现为CD62L表达升高，PD-1、LAG-3表达下降，抗原刺激后耗竭状态的CAR-T通过诱导短暂休息后，可恢复其抗肿瘤能力。

（二）免疫检查点抑制剂

程序性死亡蛋白-1（PD-1）通过与肿瘤细胞表面配体PD-L1结合诱导T细胞功能丧失及衰老凋亡，实现肿瘤细胞的免疫逃逸。PD-1/PD-L1抑制剂可阻断两者结合，恢复T细胞识别并杀伤肿瘤的功能。同时，PD-1/PD-L1抑制剂可阻断MDSC和肿瘤相关巨噬细胞（TAM）间的相互识别，可减少MDSC的免疫抑制反应，增强巨噬细胞的吞噬功能。

研究表明PD-1/PD-L1抑制剂是联合CAR-T治疗的有效靶点。Cherkassky等[10]发现回输CAR-T细胞后的小鼠体内肿瘤细胞表面PD-L1和CAR-T细胞表面PD-1表达均上调。Fraietta等[11]发现接受CAR-T细胞的CLL患者体内CD8^+^PD-1^+^ T细胞比例增加与预后不良显著相关。

目前CAR-T细胞治疗淋巴瘤的完全缓解率为52％～67％，联合治疗可将CR率提升至72.7％～79％，其有效性仍待进一步探索。在安全性方面，PD-1抑制剂不会增加CRS的发生率，但与共刺激域为CD28的CAR-T产品Axi-cel的联合治疗中出现明显增强的神经毒性。

CAR-T细胞联合PD-1抑制剂的机制包括药物联合CAR-T、CAR-T细胞自分泌抗免疫检查点分子以及免疫检查点基因编辑的CAR-T细胞。相较于基因技术，静脉注射PD-1抑制剂操作简便，可以选择最佳的剂量及应用时机。ZUMA-6[12]和PORTIA[13]研究结果显示回输后第1天开始使用PD-1抑制剂的疗效更佳，并在进一步机制探索中发现这种优化来源于CAR-T细胞杀伤和存续功能的增强而非CAR-T细胞数目的增加。因此在治疗指征明确的情况下，PD-1抑制剂应当在CAR-T细胞扩增高峰前使用，以获得更佳显著的疗效。即使如此，PD-1单抗也可用于CAR-T治疗后的挽救性用药，并取得了一定的临床获益[14]–[15]。

（三）免疫调节剂

免疫调节类药物来那度胺可以从多种机制调控CAR-T细胞活性和功能，包括增强T细胞与肿瘤细胞间的免疫突触强化杀伤功能、降低免疫检查点对T细胞的免疫抑制作用、促CD28的酪氨酸磷酸化从而激活T细胞的共刺激域、促T细胞分泌IL-21从而增加记忆性T细胞亚群等。一项临床前研究[16]表明，联合来那度胺治疗可增加CAR-T细胞在体内的存活时间。来那度胺呈剂量依赖性刺激CD8^+^T细胞亚群优先扩增，选择性增加Th1型细胞因子如IFN-γ和TNF-α的产生，减少IL-5、IL-10等免疫抑制性细胞因子的生成。通过在CAR结构上添加对来那度胺诱导降解敏感的标签，来那度胺可以重定向泛素连接酶，诱导CAR结构的泛素化和蛋白酶体的降解，成为CAR-T细胞的安全性“开关”[17]。

Li等[18]描述1例三打击R/R DLBCL患者经CAR-T治疗后2个月达到完全缓解，通过口服来那度胺维持治疗达到超过24个月的持续缓解。Thieblemont等[19]在2020年ASH上报告来那度胺应用于CAR-T治疗失败的DLBCL患者，其OR率和CR率分别为28％和16％，并且早期应用组（CAR-T输注后15 d内）具有更好的疗效（OR率为64％，CR率为36％）和更高的CAR-T细胞扩增水平。为了进一步探索来那度胺联合CAR-T治疗的可能受益人群和最佳使用时机，更多的前瞻性研究有待开展。

（四）表观遗传学药物

1. 组蛋白去乙酰化酶抑制剂（HDACi）：西达本胺（Chidamide）是一种国产新型的HDACi，其选择性地抑制HDAC1、2、3和10的活性。西达本胺通过调节T细胞表型及NK细胞、调节性T细胞（Treg）等活性达到与CAR-T细胞的协同效应，包括增加CAR-T细胞中枢记忆细胞亚群的比例、降低Treg介导的免疫抑制、调NK细胞和CD8^+^T细胞的抗肿瘤免疫的相关基因表达等。临床前研究表明，HDACi通过调节蛋白质转运通路，可以提高B7-H3[20]、CD22[21]及B7-H3-CAR在细胞表面的表达[20]，强化CAR-T细胞和肿瘤细胞的相互识别。目前在临床工作中，西达本胺可用于CAR-T细胞回输前的衔接治疗或CAR-T治疗失败后的挽救治疗，其临床疗效有待观察。

2. 去甲基化药物：地西他滨（Decitabine）通过降低肿瘤及免疫细胞相关基因甲基化水平发挥抗肿瘤作用。地西他滨可以上调淋巴瘤细胞系中CD19的表达。低剂量地西他滨与CAR-T细胞共培养后，在体外CAR-T细胞大量分化为中央记忆细胞，在没有靶细胞刺激的情况下分泌更高水平的T细胞增殖与杀伤相关细胞因子（IL-2、TNF-α、IFN-γ等）；在体内经抗原刺激后PD-1/TIM-3/LAG-3等抑制性受体表达明显低于正常CAR-T细胞，表现为低耗竭状态[22]。

2例非霍奇金淋巴瘤患者接受地西他滨、CD19 CAR-T序贯治疗后达CR，但均出现严重CRS和ICANS[23]。2021年ASH上报道预处理加入地西他滨的DFC方案，患者CR、总生存（OS）、无进展生存（PFS）均优于FC方案[24]。

（五）其他

1. 4-1BB受体激动剂：4-1BB属于肿瘤坏死因子受体超家族的重要成员，主要表达于活化的CD4^＋^和CD8^＋^T细胞，其配体表达于抗原提呈细胞。当4-1BB与抗体激动剂结合，激活的共刺激信号可促进T细胞的增殖和活化，并抑制活化诱导的细胞凋亡，增强T细胞的免疫杀伤功能[25]。4-1BB激活型抗体Utomilumab通过配体阻断途径激活CAR-T细胞，其与Axi-cel联合治疗R/R DLBCL的Ⅰ期临床试验（ZUMA-11）正处于患者招募阶段。

2. α-甲酪氨酸（MTR）：在CRS中，巨噬细胞释放大量细胞因子引发炎症级联反应，例如儿茶酚胺由巨噬细胞分泌，转而刺激巨噬细胞释放更多的细胞因子，形成正向反馈。MTR可以阻断儿茶酚胺合成限速酶酪氨酸羟化酶，破坏儿茶酚胺自我放大的环形通路，减少巨噬细胞分泌的细胞因子[26]。MTR抑制CAR-T治疗相关的CRS反应的效果已在细胞和动物实验中得到证实，其在临床患者毒性管理中的应用前景值得期待。

二、CAR-T细胞联合放疗

放疗（RT）通过对实体瘤或血液系统髓外病变的精准杀伤，可以替代化疗完成局部包块的清淋，为CAR-T细胞体内生长提供空间。通过RT降低局部巨大包块的瘤负荷可以减少CRS、肿瘤溶解综合征等不良反应的发生，提升CAR-T治疗的安全性。临床前证据表明放疗和 CAR-T 治疗在肿瘤微环境和免疫反应方面存在更多潜在的协同作用：低剂量 RT 的调理作用增强CAR-T对抗原阴性肿瘤细胞的杀伤作用；RT通过诱导主要组织相容性复合体-1表达增加和受照射细胞上抗原的释放，促进受照射部位和远处扩散部位均产生增强的肿瘤特异性免疫；RT促进细胞毒性T细胞向照射区域的迁移，逆转T细胞耗竭，并丰富肿瘤浸润淋巴细胞的T细胞受体库。

选择放疗的时机是与CAR-T细胞联合治疗的重点难点。临床数据显示淋巴细胞计数减少与放疗相关，提示进行放射疗法的理想时机应在单个核细胞采集后。多个回顾性研究[27]–[29]表明RT作为单采与CAR-T细胞回输间的桥接治疗，可以提升患者的临床疗效，降低高瘤负荷患者的治疗风险。但也有证据表明局部照射本身并不具有免疫抑制作用，并且大部分迁移至肿瘤内的T细胞可以在临床剂量的辐射下存活。Smith等[30]报道1例在CAR-T治疗后出现脊髓压迫症状，于回输后第6天开始接受放疗和大剂量激素治疗，CAR-T细胞在放疗结束后出现二次扩增，提示放疗与CAR-T可能存在远隔的协同效应。探索多病种（NCT04790747、NCT04726787、ChiCTR2100050387等）及联合时机（NCT04888338、NCT04601831等）的临床试验正在进行中。

三、CAR-T细胞联合自体造血干细胞移植（ASCT）

桥接ASCT有利于CAR-T治疗后获CR的高危淋巴瘤患者的长期存活，未缓解的淋巴瘤患者在CAR-T治疗前2～6 d进行ASCT同样可以取得相当好的疗效。早期研究表明，造血干细胞移植前的清髓性预处理化疗可清除抑制性肿瘤微环境（如Treg、MDSC），增加淋巴细胞增殖性细胞因子（IL-7、IL-15等）的释放，并在动物实验中验证其有利于CD8^+^T细胞的体内增殖。同时，移植后造血重建及免疫重建也有助于CAR-T细胞体内增殖及长期存续、保留CAR-T细胞的持续免疫监控作用。

近年来，ASCT序贯CAR-T在治疗侵袭性淋巴瘤中表现优秀[31]–[33]，可以显著改善TP53突变的高危患者的长期预后[34]，但其对CAR-T细胞的激活也可能导致治疗过程中的严重不良反应。美国纪念凯瑟琳癌症中心[31]发现给予大剂量化疗、ASCT和序贯输注CAR-T，60％（9/15）的患者出现严重的神经毒性，严重不良反应的高发生率可能与CAR-T细胞的大剂量输注（5×10^6^/kg或1×10^7^/kg）、CAR-T细胞急剧扩增相关，通过选择4-1BB共刺激域的CAR-T细胞可以减少不良反应的发生。一项ASCT序贯CD19/22 CAR-T治疗的研究[32]显示仅有5％的患者出现3级CRS和ICANS，另一项ASCT序贯CD19 CAR-T治疗的临床试验[33]中出现2例4级ICANS。为降低严重ICANS的发生，我们需要进一步加强毒性管理和探索联合机制。

四、总结

解决淋巴瘤中CAR-T治疗的耐药、复发问题是CAR-T疗法进一步发展的重要方向之一。根据患者的疾病负荷、高危因素、基础情况及回输CAR-T细胞特点合理选择方案进行联合治疗，将进一步提高CAR-T疗法的疗效及安全性。在真实世界病案报告及机制研究的基础上，CAR-T联合治疗策略仍需不断完善，特别是在个体化联合药物或治疗的剂量、时机选择方面仍需进一步明确，亟需更多严格的临床试验来证实。
